# Keep Fingers on the CpG Islands

**DOI:** 10.3390/epigenomes8020023

**Published:** 2024-06-19

**Authors:** Xing Zhang, Robert M. Blumenthal, Xiaodong Cheng

**Affiliations:** 1 Department of Epigenetics and Molecular Carcinogenesis, The University of Texas MD Anderson Cancer Center, Houston, TX 77030, USA; xzhang21@mdanderson.org; 2 Department of Medical Microbiology and Immunology, and Program in Bioinformatics, The University of Toledo College of Medicine and Life Sciences, Toledo, OH 43614, USA; robert.blumenthal@utoledo.edu

**Keywords:** CpG islands in promoters, CpG-free segments, C2H2 zinc-finger arrays, DNA sequence-specific binding, DNA methylation, epigenetic reprogramming

## Abstract

The post-genomic era has ushered in the extensive application of epigenetic editing tools, allowing for precise alterations of gene expression. The use of reprogrammable editors that carry transcriptional corepressors has significant potential for long-term epigenetic silencing for the treatment of human diseases. The ideal scenario involves precise targeting of a specific genomic location by a DNA-binding domain, ensuring there are no off-target effects and that the process yields no genetic remnants aside from specific epigenetic modifications (i.e., DNA methylation). A notable example is a recent study on the mouse *Pcsk9* gene, crucial for cholesterol regulation and expressed in hepatocytes, which identified synthetic zinc-finger (ZF) proteins as the most effective DNA-binding editors for silencing *Pcsk9* efficiently, specifically, and persistently. This discussion focuses on enhancing the specificity of ZF-array DNA binding by optimizing interactions between specific amino acids and DNA bases across three promoters containing CpG islands.

## 1. Three Primary Methods of Using DNA Binding Proteins for Epigenetic Editing

The complete sequencing of the human genome, including the heterochromatic regions and all centromeric satellite array repeats [1], has greatly accelerated the pace of locus-specific targeted engineering for epigenomic modifications. Three primary methods for epigenetic editing have emerged, enabling targeted alterations in gene expression: C2H2 zinc finger (ZF) proteins [2], transcription activator-like effectors (TALEs) [3], and enzymatic deactivated CRISPR-associated dCas9 protein [4] (reviewed in [5,6,7] and references therein). These methods have potentially profound therapeutic benefits [8] but are not without their challenges, most notably off-target activities. A recent study targeted the mouse *Pcsk9* gene, which plays a crucial role in cholesterol homeostasis, is expressed in hepatocytes, and (in humans) is associated with familial hypercholesterolemia. *Pcsk9* (Proprotein Convertase Subtilisin/kexin type 9) controls the production of cell-surface receptors of low-density lipoprotein (LDL) [9]. This study found that synthetic ZF proteins were the best-performing DNA-binding editors for efficiently silencing mouse *Pcsk9* [10]. Specifically, ZF-based engineered repressors were 5.7× and 2.8× more potent in silencing *Pcsk9* than were dCas9- and TALE-based repressors, respectively [10]. Here, we discuss optimizing the specificity of ZF—DNA interactions in order to further enhance the precision of epigenetic editing, using *Pcsk9* as the model target.

## 2. CpG Island of Mouse *Pcsk9*

**Figure 1:**
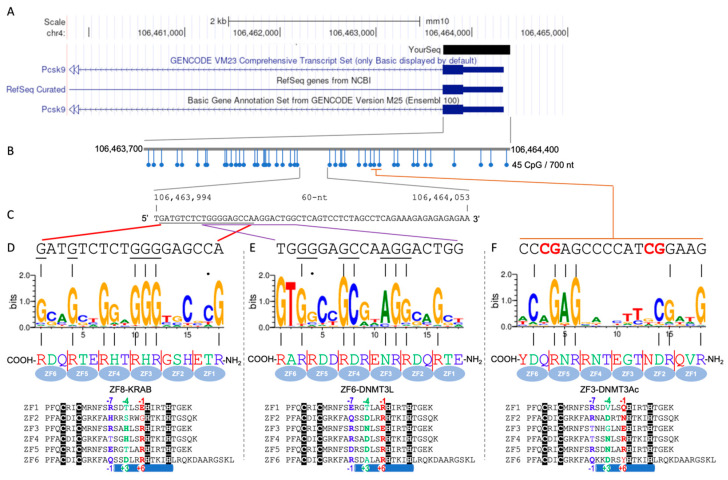
CpG island of mouse *Pcsk9* targeted by three ZF fusion proteins. (**A**) Mouse *Pcsk9* is located on chromosome 4 (mm10). (**B**) The 700 nt CGI that spans the promoter region of *Pcsk9* contains 45 CpG dinucleotides. (**C**) There is a CpG-free 60 nt gap within the CGI. (**D**–**F**) The 18 bp DNA elements potentially occupied by the fusion proteins ZF8-KRAB (**D**), ZF6-DNMT3L (**E**), and ZF3-DNMT3Ac (**F**). Top line: the actual 18 bp DNA sequence from 5′ to 3′ (left to right). Second line: sequence logo generated using a random forest (RF) prediction model [15], with regression on a bacterial one-hybrid system (B1H) [16,17,18]; the matched purines between the actual and the predicted DNA-binding sequences are indicated by vertical lines. Third line: the three base-interacting residues at −7, −4, and −1 of each finger from the NH_2_-to-COOH termini (right-to-left). The bottom section shows all six ZF motifs from each fusion protein sequence, taken from supplementary information Table 6 of [10]. The matching text colors in the third line and bottom section highlight the key recognition residues at positions −1, −4, and −7 of each finger as indicated. Note: this sequence-based numbering (−1, −4, and −7), relative to the first Zn-associated histidine, corresponds to the structure-based numbering of +6, +3, and −1 (relative to the start of the α-helix) [19].

CpG islands (CGIs) are DNA sequences that are rich in CpG dinucleotides that remain predominantly unmethylated [11] and typically found within or near gene promoters [12,13]. This characteristic has been conserved across 239 primate genomes [14], strongly implying their significance in gene regulation. In mouse *Pcsk9*, the CGI that spans the promoter region has been specifically targeted by engineered ZF proteins, each comprising an array of six zinc-finger units (Figure 1). Among the sixteen designer ZF proteins (named ZF1-16), three of them (ZF3, 6 and 8) were selected, using the efficiency of *Pcsk9* repression as a readout in an engineered mouse hepatoma cell line that reports transcriptional activity of this gene at the single-cell level [10]. These three ZF proteins were fused to different functional domains: the catalytic domain of DNMT3A (DNMT3Ac), DNMT3-like (DNMT3L), and the Krüppel-associated box (KRAB) domain [10]. The resulting fusion proteins—named ZF3-DNMT3Ac, ZF6-DNMT3L, and ZF8-KRAB—are designed, upon joint localization to a specific site, to emulate a repressive complex akin to the naturally occurring complexes mediated by KRAB-associated protein complexes.

Naturally occurring KRAB-ZF proteins are characterized by their structural organization, which consists of at least one KRAB domain located at the N-terminal and a C-terminal array of tandem ZFs that confer the ability to bind a wide variety of DNA sequences with a high specificity [20]. This specificity is critical for their role in repressing transposable elements, a function that underscores the evolutionary pressure to maintain genomic integrity and stability [21,22]. The KRAB domain plays a pivotal role in this repression mechanism by serving as an interaction partner for the KRAB-associated protein (KAP1) [23,24,25]. KAP1, in turn, orchestrates the assembly of a heterochromatin complex that includes the de novo DNA methyltransferase DNMT3A in complex with its effector protein DNMT3L [26]. This complex is instrumental in mediating transcriptional repression through both chromatin remodeling and DNA methylation.

The 700-nucleotide (nt) CGI associated with *Pcsk9* features 45 CpG dinucleotides (Figure 1B), flanking a central region with a 60 nt span devoid of any CpGs (Figure 1C). The three fusion proteins (ZF3-DNMT3Ac, ZF6-DNMT3L, and ZF8-KRAB) demonstrate distinct binding preferences within this CGI, with ZF6 and ZF8 targeting the CpG-free gap and ZF3 binding to a region situated downstream. This specificity of binding is underpinned by the nature of three amino acids within each finger (see below), where each ZF unit typically interacts with three consecutive base pairs of DNA, referred to as the “triplet” element [27,28]. Consequently, an array comprising six tandem ZF units would interact with a DNA sequence spanning 18 base pairs. To elucidate the DNA-binding specificities of each fusion protein, using their protein sequences as inputs, we generated the predicted DNA-binding specificities using a computational algorithm [29] and displayed them as sequence logos (Figure 1D–F).

We note concordance between the predicted and actual DNA-binding sequences within the CGI, but it is only partial. Specifically, for the ZF8-KRAB fusion protein, only 6 out of the 18 targeted positions match the predicted binding sites (Figure 1D). Similarly, the ZF6-DNMT3L fusion protein exhibits a match for 7 out of 18 positions (Figure 1E), while ZF3-DNMT3Ac matches at 6 out of 18 positions (Figure 1F). The matching is particularly poor for the DNA sequences corresponding to the first two ZF units of ZF8-KRAB and ZF6-DNMT3L, as well as the two central units of ZF3-DNMT3Ac (Figure 1D–F). It is possible that the six fingers do not all engage in DNA binding simultaneously, further complicating the prediction of genomic binding sites. Moreover, the binding sequences for ZF6-DNMT3L and ZF8-KRAB partially overlap, suggesting a competitive or exclusive binding scenario in which it is unlikely for both fusion proteins to bind to their target sites simultaneously due to spatial constraints. Such overlaps among naturally occurring binding proteins are known to play regulatory roles [30]. This partial overlap suggests that the prediction of DNA-binding specificities, while informative, does not fully capture the complexity of in vivo DNA–protein interactions.

The established recognition code for C2H2-ZF proteins outlines how each finger unit is capable of recognizing the 5′, central, and 3′ bases of a specific DNA base-pair triplet via base-interacting residues located at the −1, −4, and −7 positions between the last zinc-coordinating cysteine and the first zinc-coordinating histidine (see protein sequences in the bottom of Figure 1). In the context of the CGI and the engineered three ZF fusion proteins, the congruency between the predicted and actual binding sequences has been found predominantly with guanine (G) bases (Figure 1D–F). This observation is consistent with the established recognition code, where the guanines within the target sequences are primarily recognized via hydrogen bonds in the DNA major groove by the arginine (R) or histidine (H) residues present at the base-interacting positions. This specificity could be further enhanced by the broader recognition capabilities, by hydrogen bonds between guanine and lysine (K), between adenine (A) and asparagine (N) or glutamine (Q), and between cytosine (C) and aspartate (D), while thymine (T) is recognized via either C-H•••O type interactions or van der Waals contacts with glutamate (E) or hydrophobic residues [19].

## 3. Improved Specificity

Based on the ZF8-KRAB model, Cappelluti et al. designed a single ZF protein that incorporates both DNMT3Ac and DNMT3L at its N-terminus, with the KRAB domain attached at the C-terminus, resulting in a multidomain fusion protein: DNMT3Ac-DNMT3L-ZF8-KRAB [10]. This approach streamlines the delivery process by eliminating the need to co-deliver three separate mRNA molecules and also reduces the potential for off-target effects observed with the ZF3 and ZF6 fusion proteins. This “all-in-one” design strategy has seen previous applications in TALE [3] and dCas9 [4]. We suggest that further optimization of the ZF8 fusion component could enhance the efficacy and specificity. Optimization could involve refining the ZF8 base-interacting residues for greater specificity, and/or expanding it to an array of nine ZFs for a 27 bp unique sequence.

**Figure 2:**
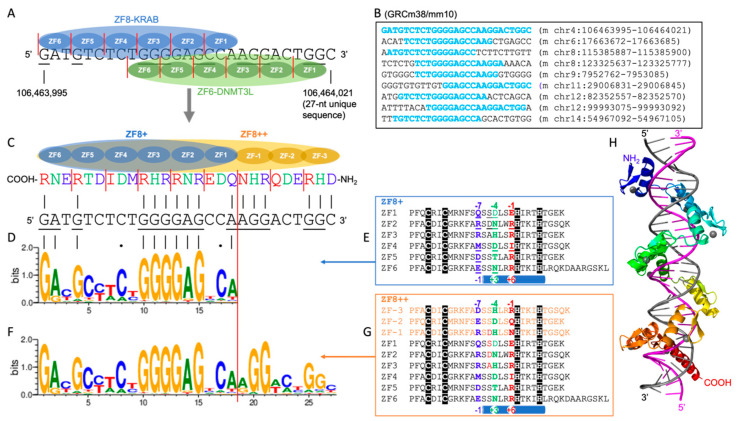
Improved specificity based on ZF8. (**A**) Overlap between ZF8-KRAB and ZF6-DNMT3L. The 18 bps recognized by ZF8-KRAB and the 18 bps recognized by ZF6-DNMT3L overlap by 10 bps. Together, they recognize a 27 bp segment. (**B**) Several shorter sequences, under 27 nt, display partial matches on other chromosomes of the mouse genome (GRCm38/mm10). (**C**) The design of an expanded nine-finger protein. The protein sequence from the NH_2_ to COOH termini (right-to-left) runs antiparallel to that of the DNA sequence from the 5′ to 3′ ends (left-to-right). (**D**,**E**) Improved specificity of ZF8^+^ fusion protein (sequence logo in (**D**)) and the corresponding protein sequence with altered residues underlined (**E**). (**F**,**G**) Improved specificity of ZF8^++^ fusion protein (sequence logo in (**F**)) and the corresponding protein sequence of the nine-finger array (**G**). Note that the sequence-based numbering (−1, −4, and −7) and the structure-based numbering (+6, +3, and −1) are provided above and below the sequences, respectively. (**H**) An AlphaFold3 prediction of ZF8^++^ in a complex with DNA with the nine ZF units (colored from blue to red), and the DNA recognition strand (magenta).

The overlap between ZF8-KRAB and ZF6-DNMT3L spans a 27 bp DNA segment (Figure 2A), resulting in a unique sequence on chromosome 4 of the mouse genome (GRCm38/mm10) (Figure 2B). Several shorter sequences, under 27 bp, display partial matches on other chromosomes (Figure 2B). We then made two optimizations (Figure 2C). First, we refined the amino acid composition at the three-base interaction sites for each finger within the ZF8-fusion protein—specifically, at the −4 and −7 positions of ZF1, the −1, −4, and −7 positions of ZF2 and ZF4, the −7 position of ZF5, and the −4 and −7 positions of ZF6. This optimization yielded the ZF8^+^ fusion, having a perfect alignment of 10 purines (G and A) and two cytosines (Figure 2D,E). Following this, we extended the array at the N-terminus by three additional fingers, creating a nine-finger array (ZF8^++^ fusion) tailored for the 27 bp DNA sequence (Figure 2F,G).

We note that, in earlier studies, designed or selected three-finger proteins were shown to display sufficient affinity and specificity to act at nine-base-pair recognition sites in vivo (reviewed in [28]). However, several studies found that four, five, or six linked fingers, or even a nine-finger protein, displayed only modest improvements in affinity over the three-finger constructs (ref. [28] and references therein). This can be understood if the additional fingers did not provide specificity outside of the nine-base-pair recognition site. More recent studies revealed that five or six-finger PRDM9 [31,32], 11-finger CTCF [33], and 11-finger ZFP568 [34] proteins can bind longer specific sequences, including DNA conformation-induced adaptable binding. A model generated by AlphaFold3 [35] of nine-finger ZF8^++^ binding in the DNA major groove indicated that it follows the right-handed twist of the 27-base-pair DNA in a canonical manner (Figure 2H).

## 4. CGI Islands of Mouse *Ldlr* and *Ankrd26*

**Figure 3:**
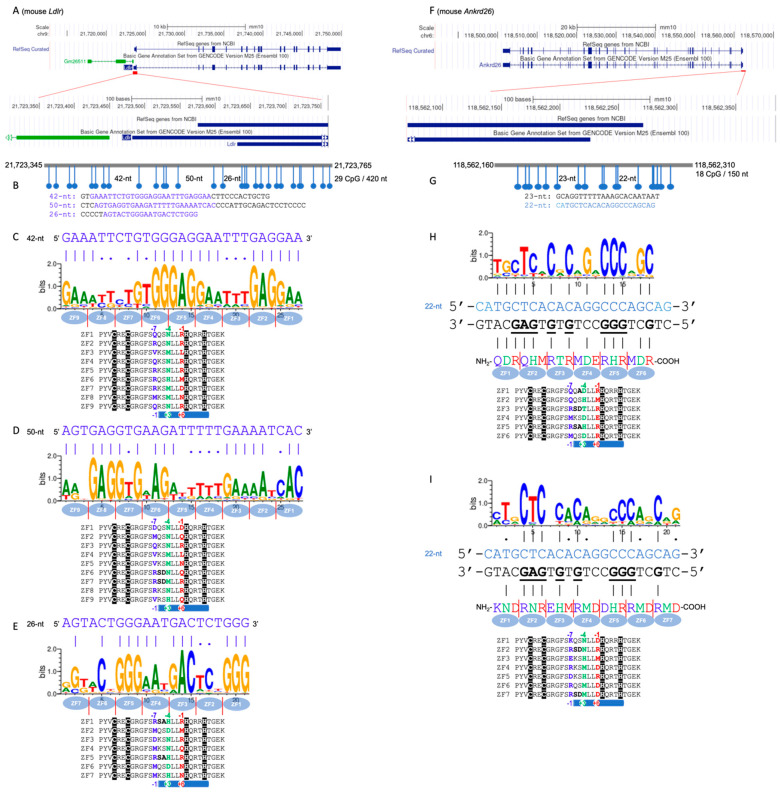
CGIs of mouse *Ldlr* or *Ankrd26*. (**A**) Mouse *Ldlr* is located on chromosome 9. (**B**) The 420 nucleotides of CGI that span the promoter region of *Ldlr* contain 29 CpG dinucleotides, with 3 CpG-free gaps. (**C**–**E**) Examples of three designer ZF arrays based on the backbone of PRDM9 could potentially bind the CpG-free gaps of 42 nt, 50 nt, or 26 nt. Sequence logo generated by a prediction model and the matched purines (G and A) and cytosines of the actual sequence (top) are indicated by vertical lines. (**F**) Mouse *Ankrd26* is located on chromosome 6. (**G**) The smaller CGI contains CpG-free gaps of 22 or 23 nt. (**H**,**I**) Examples of two designer ZF arrays that could potentially bind the guanine-rich strand of the 22 nt gap. Note that the sequence-based numbering (−1, −4, and −7) and the structure-based numbering (+6, +3, and −1) are provided above and below the sequences, respectively.

Another recent study by Takahashi et al. (2023) explored the methylation of CGIs in two mouse genes that are critical to metabolism: the low-density lipoprotein receptor (*Ldlr*) and ankyrin repeat domain 26 (*Ankrd26*) [36]. Disabling *Ldlr* or *Ankrd26* leads to hypercholesterolemia or obesity, respectively, without impacting mouse survival or reproductive capacity [37,38]. Takahashi et al. inserted a 4.3 kb CpG-free fragment into the relatively compact CGIs of *Ldlr* (420-nt) and *Ankrd26* (150-nt) (Figure 3). This insertion diluted the CpG dinucleotide density and triggered CGI methylation in mouse embryonic stem (mES) cells. Following the removal of the CpG-free fragment through genetic engineering [39], leaving a small genetic alteration within the CGI, the modified mES cells were introduced into eight-cell mouse embryos. Notably, the resulting DNA methylation patterns were stable in adult mice and were heritable over at least four generations. While that study primarily investigated the mechanisms of transgenerational epigenetic inheritance [40,41], our commentary focuses on the induction of de novo DNA methylation at previously unmethylated CGIs using ZF fusion proteins.

For the *Ldlr* CGI, three CpG-free intervals are identified, spanning 42 nt, 50 nt, and 26 nt (Figure 3A,B). Each interval features a purine-rich strand, which can be targeted by either a nine- or seven-finger array, detailed in Figure 3C–E. Our array design draws inspiration from PRDM9 [31,32], notable among ZF proteins for its highly repetitive fingers, derived through sequence duplications. This characteristic enables the fine-tuning of nearly identical fingers, distinguished only by amino acid variations at positions interacting with the DNA bases, to accommodate sequence variability in the target DNA. In the case of the *Ankrd26* CGI, this smaller CGI, measuring 150 nt, encompasses two CpG-free regions of 23 nt and 22 nt (Figure 3F,G). For the 22 nt gap, which is guanine-rich, we designed a targeting array comprising six or seven fingers, specifically aiming at the guanine-rich sequence within this gap (Figure 3H,I).

## 5. Concluding Remarks

**Figure 4:**
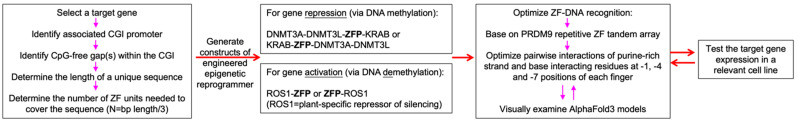
Flowchart of stepwise approach for producing ZF-based engineered epigenetic reprogrammers. ROS1 is a plant-specific repressor of silencing 1 [42].

To develop an effective epigenetic editing tool, the precision of the DNA-binding domain is crucial and generally requires a recursive process (Figure 4). The recognition of longer DNA sequences increases the likelihood of identifying a unique sequence. The modular nature of the C2H2 ZF unit enables the creation of an array of fingers that can recognize these extended sequences. However, the number of fingers alone does not guarantee specificity. For example, CTCF, which has eleven tandem fingers, typically uses only 4–5 of these fingers to bind a 12–15-base-pair core sequence among tens of thousands of potential sites on mammalian chromosomes (ref. [33] and references therein). In contrast, the 11-finger mouse Zfp568 specifically binds a 24 nt motif located upstream of the Igf2-P0 promoter [34]. The key challenge is ensuring that each finger engages the DNA simultaneously to enhance binding precision.

Seven large, charged, or polar residues—Arg, His, Lys, Asn, Gln, Asp, and Glu—play key roles in DNA base-specific interactions within the major groove. Such interactions are especially significant when these residues are situated at three specific locations in C2H2-ZF proteins (−1, −4, −7 relative to the first Zn-coordinating His) to enable precise interactions with three consecutive base pairs (one finger–three base rule). The placement of these residues at the base-interacting positions imparts sequence specificity to one strand of the double-stranded DNA. Targeting the purine-rich strand (G and A) is eased by pairing G with Arg, His, or Lys, and A with Asn or Gln. Deviations from the one finger–three base rule are known, such as interacting with just two bases, which can sufficiently secure a finger’s grip on the DNA. This anchoring of the DNA by fingers at the N- or C-terminal ends of the protein is particularly crucial to ensure that every intermediate finger engages the DNA simultaneously.

Additional considerations involve small and non-aromatic hydrophobic residues at base-interacting positions, which often provide “versatile” contacts that can enhance binding affinity. In some cases, these residues engage in C-H•••O interactions or van der Waals interactions with the methyl group of thymine in A/T-rich sequences. To fully leverage the determinants guiding C2H2-ZF fingers to bind within the DNA major groove, further refinement at other positions is essential. This includes inducing DNA conformational changes upon binding and facilitating cross-strand interactions to enhance specificity [19].

## References

[1] Nurk S., Koren S., Rhie A., Rautiainen M., Bzikadze A.V., Mikheenko A., Vollger M.R., Altemose N., Uralsky L., Gershman A. (2022). The complete sequence of a human genome. Science.

[2] Paschon D.E., Lussier S., Wangzor T., Xia D.F., Li P.W., Hinkley S.J., Scarlott N.A., Lam S.C., Waite A.J., Truong L.N. (2019). Diversifying the structure of zinc finger nucleases for high-precision genome editing. Nat. Commun..

[3] Mlambo T., Nitsch S., Hildenbeutel M., Romito M., Muller M., Bossen C., Diederichs S., Cornu T.I., Cathomen T., Mussolino C. (2018). Designer epigenome modifiers enable robust and sustained gene silencing in clinically relevant human cells. Nucleic Acids Res..

[4] Nunez J.K., Chen J., Pommier G.C., Cogan J.Z., Replogle J.M., Adriaens C., Ramadoss G.N., Shi Q., Hung K.L., Samelson A.J. (2021). Genome-wide programmable transcriptional memory by CRISPR-based epigenome editing. Cell.

[5] Chandrasegaran S., Carroll D. (2016). Origins of Programmable Nucleases for Genome Engineering. J. Mol. Biol..

[6] Waryah C.B., Moses C., Arooj M., Blancafort P. (2018). Zinc Fingers, TALEs, and CRISPR Systems: A Comparison of Tools for Epigenome Editing. Methods Mol. Biol..

[7] Mazloum A., Karagyaur M., Chernyshev R., van Schalkwyk A., Jun M., Qiang F., Sprygin A. (2023). Post-genomic era in agriculture and veterinary science: Successful and proposed application of genetic targeting technologies. Front. Vet. Sci..

[8] Cortes-Mancera F.M., Sarno F., Goubert D., Rots M.G. (2022). Gene-Targeted DNA Methylation: Towards Long-Lasting Reprogramming of Gene Expression?. Adv. Exp. Med. Biol..

[9] Seidah N.G., Prat A. (2022). The Multifaceted Biology of PCSK9. Endocr. Rev..

[10] Cappelluti M.A., Mollica Poeta V., Valsoni S., Quarato P., Merlin S., Merelli I., Lombardo A. (2024). Durable and efficient gene silencing in vivo by hit-and-run epigenome editing. Nature.

[11] Bird A.P. (1986). CpG-rich islands and the function of DNA methylation. Nature.

[12] Ioshikhes I.P., Zhang M.Q. (2000). Large-scale human promoter mapping using CpG islands. Nat. Genet..

[13] Weber M., Hellmann I., Stadler M.B., Ramos L., Paabo S., Rebhan M., Schubeler D. (2007). Distribution, silencing potential and evolutionary impact of promoter DNA methylation in the human genome. Nat. Genet..

[14] Kuderna L.F.K., Ulirsch J.C., Rashid S., Ameen M., Sundaram L., Hickey G., Cox A.J., Gao H., Kumar A., Aguet F. (2024). Identification of constrained sequence elements across 239 primate genomes. Nature.

[15] Breiman L. (2001). Random forests. Mach. Learn..

[16] Persikov A.V., Rowland E.F., Oakes B.L., Singh M., Noyes M.B. (2014). Deep sequencing of large library selections allows computational discovery of diverse sets of zinc fingers that bind common targets. Nucleic Acids Res..

[17] Persikov A.V., Wetzel J.L., Rowland E.F., Oakes B.L., Xu D.J., Singh M., Noyes M.B. (2015). A systematic survey of the Cys2His2 zinc finger DNA-binding landscape. Nucleic Acids Res..

[18] Najafabadi H.S., Mnaimneh S., Schmitges F.W., Garton M., Lam K.N., Yang A., Albu M., Weirauch M.T., Radovani E., Kim P.M. (2015). C2H2 zinc finger proteins greatly expand the human regulatory lexicon. Nat. Biotechnol..

[19] Zhang X., Blumenthal R.M., Cheng X. (2024). Updated understanding of the protein-DNA recognition code used by C2H2 zinc finger proteins. Curr. Opin. Struct. Biol..

[20] Urrutia R. (2003). KRAB-containing zinc-finger repressor proteins. Genome Biol..

[21] Seah M.K.Y., Wang Y., Goy P.A., Loh H.M., Peh W.J., Low D.H.P., Han B.Y., Wong E., Leong E.L., Wolf G. (2019). The KRAB-zinc-finger protein ZFP708 mediates epigenetic repression at RMER19B retrotransposons. Development.

[22] Wolf G., de Iaco A., Sun M.A., Bruno M., Tinkham M., Hoang D., Mitra A., Ralls S., Trono D., Macfarlan T.S. (2020). KRAB-zinc finger protein gene expansion in response to active retrotransposons in the murine lineage. eLife.

[23] Friedman J.R., Fredericks W.J., Jensen D.E., Speicher D.W., Huang X.P., Neilson E.G., Rauscher F.J. (1996). KAP-1, a novel corepressor for the highly conserved KRAB repression domain. Genes Dev..

[24] Ozato K., Shin D.M., Chang T.H., Morse H.C. (2008). TRIM family proteins and their emerging roles in innate immunity. Nat. Rev. Immunol..

[25] Stoll G.A., Pandiloski N., Douse C.H., Modis Y. (2022). Structure and functional mapping of the KRAB-KAP1 repressor complex. EMBO J..

[26] Jia D., Jurkowska R.Z., Zhang X., Jeltsch A., Cheng X. (2007). Structure of Dnmt3a bound to Dnmt3L suggests a model for de novo DNA methylation. Nature.

[27] Choo Y., Klug A. (1997). Physical basis of a protein-DNA recognition code. Curr. Opin. Struct. Biol..

[28] Wolfe S.A., Nekludova L., Pabo C.O. (2000). DNA recognition by Cys2His2 zinc finger proteins. Annu. Rev. Biophys. Biomol. Struct..

[29] Persikov A.V., Singh M. (2014). De novo prediction of DNA-binding specificities for Cys2His2 zinc finger proteins. Nucleic Acids Res..

[30] Ngondo-Mbongo R.P., Myslinski E., Aster J.C., Carbon P. (2013). Modulation of gene expression via overlapping binding sites exerted by ZNF143, Notch1 and THAP11. Nucleic Acids Res..

[31] Patel A., Horton J.R., Wilson G.G., Zhang X., Cheng X. (2016). Structural basis for human PRDM9 action at recombination hot spots. Genes Dev..

[32] Patel A., Zhang X., Blumenthal R.M., Cheng X. (2017). Structural basis of human PR/SET domain 9 (PRDM9) allele C-specific recognition of its cognate DNA sequence. J. Biol. Chem..

[33] Yang J., Horton J.R., Liu B., Corces V.G., Blumenthal R.M., Zhang X., Cheng X. (2023). Structures of CTCF-DNA complexes including all 11 zinc fingers. Nucleic Acids Res..

[34] Patel A., Yang P., Tinkham M., Pradhan M., Sun M.A., Wang Y., Hoang D., Wolf G., Horton J.R., Zhang X. (2018). DNA conformation induces adaptable binding by tandem zinc finger proteins. Cell.

[35] Abramson J., Adler J., Dunger J., Evans R., Green T., Pritzel A., Ronneberger O., Willmore L., Ballard A.J., Bambrick J. (2024). Accurate structure prediction of biomolecular interactions with AlphaFold 3. Nature.

[36] Takahashi Y., Morales Valencia M., Yu Y., Ouchi Y., Takahashi K., Shokhirev M.N., Lande K., Williams A.E., Fresia C., Kurita M. (2023). Transgenerational inheritance of acquired epigenetic signatures at CpG islands in mice. Cell.

[37] Ishibashi S., Brown M.S., Goldstein J.L., Gerard R.D., Hammer R.E., Herz J. (1993). Hypercholesterolemia in low density lipoprotein receptor knockout mice and its reversal by adenovirus-mediated gene delivery. J. Clin. Investig..

[38] Bera T.K., Liu X.F., Yamada M., Gavrilova O., Mezey E., Tessarollo L., Anver M., Hahn Y., Lee B., Pastan I. (2008). A model for obesity and gigantism due to disruption of the *Ankrd26* gene. Proc. Natl. Acad. Sci. USA.

[39] Takahashi Y., Wu J., Suzuki K., Martinez-Redondo P., Li M., Liao H.K., Wu M.Z., Hernandez-Benitez R., Hishida T., Shokhirev M.N. (2017). Integration of CpG-free DNA induces de novo methylation of CpG islands in pluripotent stem cells. Science.

[40] McGraw S., Kimmins S. (2023). Inheritance of epigenetic DNA marks studied in new mouse model. Nature.

[41] Horsthemke B., Bird A. (2023). Loss of CpG island immunity to DNA methylation induced by mutation. Epigenetics Chromatin.

[42] Du X., Yang Z., Xie G., Wang C., Zhang L., Yan K., Yang M., Li S., Zhu J.-K., Du J. (2023). Molecular basis of the pant ROS1-medicated active DNA demethylation. Nat. Plants.

